# Prevalence of ATP7B Gene Mutations in Iranian Patients With Wilson Disease

**DOI:** 10.5812/kowsar.1735143X.762

**Published:** 2011-11-30

**Authors:** Narges Zali, Seyed Reza Mohebbi, Sahar Esteghamat, Mohsen Chiani, Mahdi Montazer Haghighi, Seyed Mohammad-Kazem Hosseini-Asl, Faramarz Derakhshan, Amir-Houshang Mohammad-Alizadeh, Seyed-Ali Malek-Hosseini, Mohammad Reza Zali

**Affiliations:** 1 Research Centre for Gastroenterology and Liver Diseases, Shahid Beheshti University of Medical Sciences, Tehran, Iran

**Keywords:** Wilson Disease Protein, Mutation, ATP7B Protein

## Abstract

**Background:**

Wilson disease (WD) is an autosomal recessive disorder. The WD gene, ATP7B, encodes a copper-transporting ATPase involved in the transport of copper into the plasma protein ceruloplasmin and in excretion of copper from the liver. ATP7B mutations cause copper to accumulate in the liver and brain.

**Objectives:**

We examined the ATP7B mutation spectrum in Wilson disease patients in Iran.

**Patients and Methods:**

Genomic DNA was extracted from patients with Wilson disease. The entire coding region of the ATP7B gene was amplified using PCR and analyzed using direct sequencing.

**Results:**

We identified five novel mutations in 5 Iranian patients with Wilson disease. The first was a transversion, c.2363C > T, which led to an amino acid change from threonine to isoleucine. The second mutation was a deletion, c.2532delA (Val845Ser), which occurred in exon 10. The third mutation was a transition mutation, c.2311C > G (Leu770Leu), which occurred in the TM4 domain of the ATP7B protein. The fourth mutation was a transversion, (c.3061G > A) (Lys1020Lys), in exon 14. Lastly, we identified a transversion, c.3206C > A (His1069Asn) in exon 14 which led to a change in function of the ATP loop domain of the ATP7B protein. The H1069Q mutation was identified as the most common mutation in our study population.

**Conclusions:**

Based on our findings, the H1069Q may be a biomarker that can be used in a rapid detection assay for diagnosing WD patients

## 1. Background

Wilson disease (WD, MIM #277900) is an autosomal recessive disorder of copper metabolism resulting in toxic copper accumulation in the liver and other organs such as the brain, kidney, and cornea. Clinically, increased copper in cells can result in hepatic cirrhosis and neurological damage. The prevalence of WD varies from 1/30,000 to 1/100,000 in different populations, with an estimated carrier rate of 1 in 90 [1][2]. The WD gene, which maps to chromosome 13q14.3, encodes a 165 kDa copper-transporting P-type ATPase, ATP7B (MIM #606882) [3]. ATP7B consists of 21 exons spanning a genomic region of approximately 80 kb. The protein is made up of 1465 amino acids and is predominantly expressed in the liver, with reduced expression in the brain, kidney, and placenta [4][5][6]. ATP7B has several membrane-spanning domains, including 6 copper binding domains, 8 transmembrane domains (Tm), a transduction domain that uses energy from ATP hydrolysis for cation transport, a Tm cation domain, and a phosphorylation domain [7][8]. ATP7B mutations result in copper accumulation in the liver and brain, resulting in clinical manifestations such as liver disease, hemolysis, renal alterations, and neuropsychiatric disease. Kayser-Fleischer (KF) rings in the periphery of the cornea and copper abnormalities in serum and urinary tests are frequently observed [9][10]. More than 270 different mutations have been identified in the ATP7B gene along the length of the coding region as well as in the promoter [11].

## 2. Objectives

The aim of this study was to identify mutations in a large group of Iranian patients with WD. This is the first comprehensive mutation analysis of the Iranian population

## 3. Patients and Methods

A total of 70 WD patients, 40 males and 30 females, ranging in age from 5-40 years from 54 families were included in the study. Patients were diagnosed based on clinical symptoms. Hepatic patients showed liver involvement. Neurological patients showed neurological symptoms such as tremor, clumsiness, dysarthria, and ataxia in addition to Dystonic-Parkinsonian Syndrome. Mixed-type patients were diagnosed based on liver involvement and neurological features. Criteria for diagnosing patients with WD included serum ceruloplasmin levels of 20 mg/dL, urine copper > 40 mmol/24 h, liver copper > 250 µg/g dry weight, and the presence of Kayser-Fleischer (K-F) rings. Patients who fulfilled 2 of these criteria were included in the study. Patients who were positive for hepatitis C virus (HCV), hepatitis B surface antigen (HBsAg), or hepatitis B core antibody (HBCAb), or had acute hepatitis or malignant tumors were excluded. This investigation was approved by the local Research Center for Gastroenterology and Liver Diseases (RCGLD) ethics committee and appropriate informed consent was obtained from all patients and their families.

### 3.1. Polymerase Chain Reaction and Sequencing

DNA was extracted from whole blood using standard phenol-chloroform extraction [12] . ATP7B gene exons were amplified using PCR; 35 cycles, including a 30-s denaturing step at 94°C, a 30-s annealing step at the appropriate temperature, and a 35-s extension step at 72°C, were used for amplification. The reaction mixture contained 100 ng genomic DNA as the template, 19.4 µL dH2O, 0.5 µL 10 mM dNTP, 0.75 µL 50 mM MgCl2, 12.5 pM of each primer, and 1 unit of Taq DNA polymerase (Gibco BRL, Invitrogen, Carlsbad, CA, USA) in a total volume of 25 µL. Primers were designed using Gene Runner and Primer3 software. Bi-directional PCR-sequencing was carried out using the ABI 3130xl Genetic Analyzer (Applied-Biosystems, Foster City, CA, USA). Sequences were aligned and analyzed using ClustalX2 and BioEdit software, respectively. To detect novel mutations and polymorphisms in the gene, 50 control samples were compared with samples from diseased patients. Changes were classified as polymorphisms if they did not change the amino acid sequence or when they were also detected in normal chromosomes. Based on mutations recorded at http://www.HGMD.org/, the detected mutations were named using the sequence with the GenBank accession number NM_00053.1 as a reference.

## 4. Results

**Figure 1:**
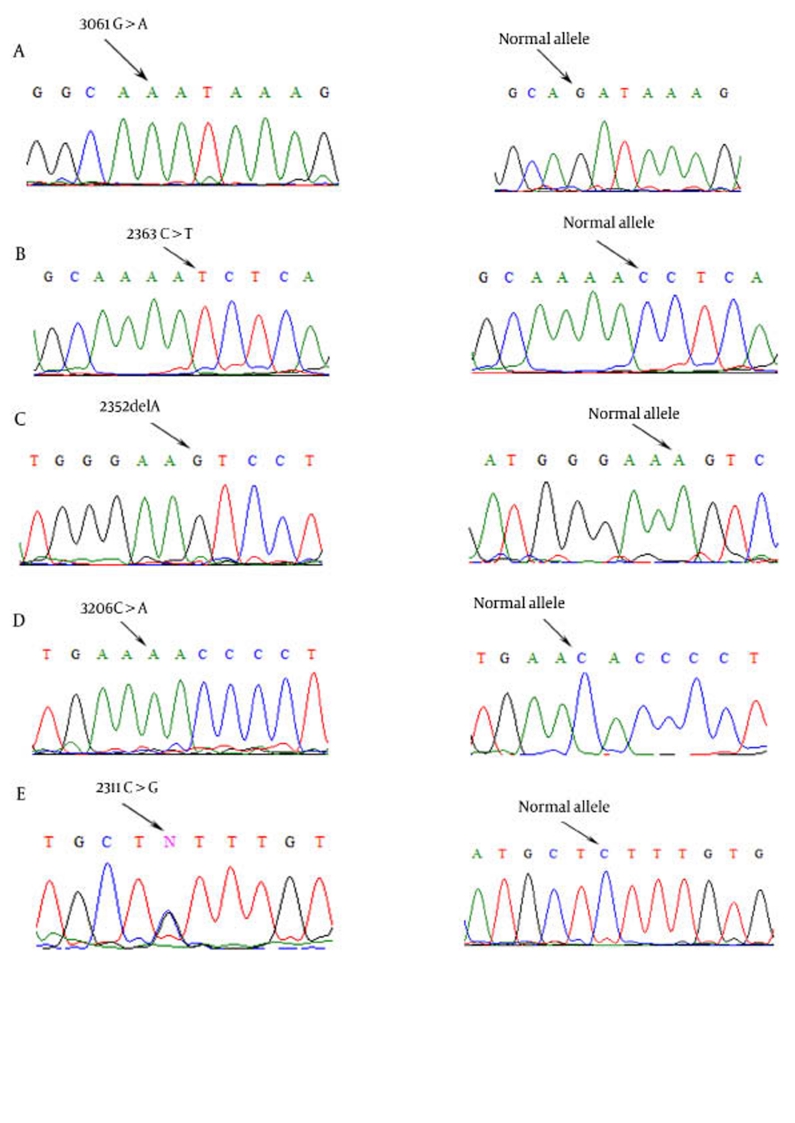
Sequencing Result for five Novel Mutations: A) c.3061G > A (K1020K) in exon 14, B) c.2363C > T (T787I) in exon 9, C) c.2532delA in exon10, D) c.3206C > A (H1069N) in exon 14 , E) c2311C > G(L770L) in exon 8

The clinical characteristics of 70 patients with WD were evaluated. Of the 70 patients, 21 (22.8%) had at least 1 mutation. Among the 21 patients with mutations, age at diagnosis ranged from 7 to 25 years (Table 1). A total of 21 different mutations were identified. According to recent publications and the "Wilson disease mutation database" (http://www.uofa-medical-genetics.org/wilson/index.html), we identified 5 novel mutations that affect various protein domains of the ATP7B gene; these mutations include c.2363C > T, c.2532delA, c.2311C > G, c.3061G > A, and c.3206C > A (Table 2,Figure 1).

**Table 1:** Characteristics of Mutations and Individual Phenotype

**Mutation**	**Nucleotide Change**	**Type**	**Exon**	**Domain**	**Manifestation**	**KF aRing (+/-)**	**Age at Onset of Symptoms**
G85V	254G > T	Missense	2	Cu1 (CuBD)	H a	_	10
845delT	845delT	Deletion	2	CuBD	H	+	7
1639delAT	1639delAT	Deletion	6	Between CuBD and TM	H	+	9
D642H	1924G > C	Missense	6	Cu6/TM1	H/N a	_	16
P840L	2519C > T	Missense	10	TD	H	_	25
V890M	2668G > A	Missense	11	TD/TM5	H	_	19
H1069Q	3207C > A	Missense	14	ATP loop			4
A874V	2621C > T	Missense	11	TD	H	_	12
A874V	2621C > T	Missense	11	TD	H	_	14
H1069Q	3207C > A	Missense	14	ATP loop	H	+	17
H1069Q	3207C > A	Missense	14	ATP loop	H	+	18
H1069Q	3207C > A	Missense	14	ATP loop	H	+	18
L1120X	3359T > A	Nonsense	15	ATP loop	H	+	11
N1270S	3809A > G	Missense	18	ATP hinge	H/N	+	17
N1270S	3809A > G	Missense	18	ATP hinge	N	_	17
R778L	2333G > T	Missense	8	TM4	H	+	22
R969Q	2906G > A	Missense	13	TM6	H	+	22

^a^ Abbreviations: H, hepatic symptom: kayser-fleisher N, neurologic symptom; KF

**Table 2:** Characteristics of 5 Novel Mutations

**Patient ID**	**Nucleotide**	**Accession No.**	**Nucleotide Change **a	**Type**	**Exon**	**Affected Protein Domain**
869710, 869711	c.2363C > T	EU041762	p.Thr787Ile	Missense	9	TM4
869171	c.2532delA	EF643605	p.Val845Ser	Deletion	10	TD
869911	c.2311C > G	EF643606	p.Leu770Leu	Silent	8	TM4
869176	c.3061G > A	EF643604	p.Lys1020Lys	Silent	14	ATP loop
869346	c.3206C > A	EF620914	p.His1069Asn	Missense	14	ATP loop

^a^ Numbering based on cDNA sequence, position +1 corresponds to the A of the ATG translation initiation codon in reference sequence; ATP7B [GenBank:NM 00053.1]

Table 3shows that a novel substitution in exon 9 involving the protein transmembrane domain at Thr787Ile was identified in 2 homozygous patients who were 23- and 28-year-old sisters; interestingly, the older sister (28 years) exhibited only neurologic manifestations, while the younger (23 years) presented with acute hepatic failure and had undergone liver transplantation. Both exhibited the KF ring. The second novel mutation identified in this study was a deletion in exon 10 (c.2532delA) in a 9-year old girl presenting with hepatic failure and neurological features. She also showed the KF ring. The third novel mutation, 2311C > G, was identified in a 22-year-old man, who demonstrated hepatic and neurologic features accompanied by the KF ring. The fourth novel mutation, c.3061G > A, was a silent mutation identified in exon 14. This mutation was found in a 14-year-old boy who also exhibited the KF ring. The fifth novel mutation, c.3206C > A, was detected in a hot spot region of exon 14. It was detected in a 21-year old girl presenting with acute hepatic failure and neurological manifestations. Additionally, she exhibited the KF ring. A total of 20 (28.6%) patients with mutations were homozygous, while one patient (1.4%) had a compound heterozygous mutation. The most commonly observed mutation was the H1069Q point mutation in 4 of 21 patients (19%), all of whom were homozygous. Interestingly, the D642H mutation was detected in members of the same family. This mutation resulted in both hepatic and neurological symptoms. Two sisters with hepatic WD were heterozygous for this mutation, while their brother was homozygous for D642H. He exhibited neurological symptoms of WD. Thus, the D642H mutation has been detected in one family and exhibited both hepatic and mixed WD presentations.

**Table 3:** Clinical Features of Individuals With Novel Mutations

**Patient ID**	**Age at Onset of Symptoms**	**Sex**	**Hepatic Failure**	**Neurological Manifestation**	**KFR a**
869710	23	Female	+	_	+
869711	28	Female	_	+	+
869171	9	Female	+	_	+
869911	22	Male	+	+	+
869176	14	Male	+	+	+
869346	21	Female	+	+	+

^a^ Abbreviation: KFR, kayser-fleisher ring

Table 2summarizes the mutations identified and corresponding ages at disease onset, presence of the KF ring, and involvement of the liver, brain, or both. In addition to the 21 different mutations, 6 different sequence variations with no clinical significance were identified (Table 4). These variants were identified in previous studies as polymorphisms detected in a normal population or as those not altering amino acid composition of the protein. To clarify whether these nucleotide and amino acid sequence changes were mutations or polymorphisms, we analyzed 50 normal individuals using direct sequencing of their PCR products.

**Table 4:** Comparison of Observed Polymorphisms in WD Patients and Control Patients

**SNP**	**Exon**	**Frequency in Cases**	**Frequency in Controls**
T1216G (A405S)	2	0.30	0.32
G1367C (L456V)	3	0.23	0.35
A2495G (K831R)	10	0.31	0.38
G2855A (K952R)	12	0.26	0.31
G3009A (A1003A)	13	0.1	0.15
G2973A (T991T)	13	0.05	0.08

## 5. Discussion

In this study, we examined the mutation spectrum of the ATP7B gene among the Iranian population. We identified 21 different mutations, including 5 novel, disease-causing mutations in 21 individuals in 70 referred patients, reflecting the genetic heterogeneity of Wilson disease. Population-frequency values of these mutations revealed that the spectrum of WD mutations consists of a large number of rare mutations. Six single nucleotide polymorphisms (SNPs) were identified (Table 4). An interesting case of marked clinical heterogeneity within a single family included 2 sisters with the homozygous T787I mutation. One exhibited only neurological manifestations, while the other presented with acute hepatic failure and had undergone liver transplantation. This considerable phenotypic variation for the same genotype suggests involvement of additional genetic, epigenetic, or even environmental factors in the pathogenesis of Wilson disease. The pathogenicity of the novel missense mutation T787I was evaluated for 3 primary factors. First, segregation analysis was performed using variable SNPs within the ATP7B gene in this family, which confirmed the presence of a mutation in the ATP7B gene responsible for causing WD. Second, T787I was not detected among 50 unrelated control subjects screened. Third, a mutation is presumed to be pathologic when it is located in the conserved region of a gene. However, the change in a hydrophilic amino acid, threonine, to a non-polar hydrophobic amino acid, isoleucine, in a conserved transmembrane domain of a protein can change its function. Further investigation is necessary to elucidate the details of this abnormal function. The most frequently observed mutation in the ATP7B gene responsible for causing WD in the U.S. and in central Europe is H1069Q, which is located in exon 14. For instance, this mutation occurs at frequencies of 61% among Austrian, 39% among Russian, 38% among North American and Swedish, 34% among Dutch, 28% among Canadian, 17% among Turkish, and 12% among Italian Wilson patients. It is associated with CNS diseases and a later onset of disease [5][13][14]. However, we observed this mutation in four (19%) patients, all of whom had only hepatic symptoms and were 5-40 years of age. This mutation disrupts the unique SEHPL amino acid motif and leads to a deficiency in ATP binding [6]. The 5 novel mutations included missense, silent, and deletion mutations. One was a novel causative mutation, H1069N. We screened 50 unrelated individuals for this mutation; none of the tested subjects was a heterozygote or a homozygote. The H1069N mutation occurs in a conserved region, and involves the conversion of the non-polar amino acid histidine to the hydrophilic amino acid asparagine within the highly-conserved SEHPL domain; alterations in this region are highly deleterious [15][16]. The Thr787Ile substitution replaces a polar residue with a non-polar and hydrophobic amino acid. This mutation is expected to change the function of the transmembrane domain by altering secondary structure. The c.2532delA is a deletion of a nucleotide (A) in exon 10; however, this mutation is silent

Mutations 2311C > G and 3060G > A were also silent; thus, no alteration in amino acid sequence was observed. R778L is one of the most prevalent mutations in Eastern Asian patients and leads to liver involvement in WD patients [17][18][19][20] .We observed this mutation in a compound heterozygous state with R969Q in 1 patient with hepatic failure. This finding may be related to the mixed ethnic background of the Iranian population. H1069Q is the most commonly observed mutation responsible for WD in our patients. This finding highlights the importance of ATP7B mutation screening in high-risk consanguineous marriages. The significance of the various mutations remains to be elucidated, and we could not identify clear genotype-phenotype associations; thus, further studies are needed. In conclusion, although mutations were distributed among different exons of the ATP7B gene in Iranian patients with WD, 6 of 21 mutations were located in exon 14. Therefore, this exon may be a mutational hot spot in the Iranian population. This finding is compatible with the results from Western countries and in contrast with results from China. Hot spot exons reported in Western countries were 14 and 18, while the hot spot exons reported in China were 8 and 12 [20]. These results are important for establishing a rapid and accurate mutation analysis method for diagnosing WD patients in the Iranian population.
